# The effect of alkyl chain and electronegative atoms in anion on biological activity of anilinium carboxylate bioactive ionic liquids and computational approaches by DFT functional and molecular docking

**DOI:** 10.1016/j.heliyon.2021.e07509

**Published:** 2021-07-08

**Authors:** Ajoy Kumer, Md. Wahab Khan

**Affiliations:** Organic Research Laboratory, Department of Chemistry, Bangladesh University of Engineering and Technology (BUET), Dhaka, 1000, Bangladesh

**Keywords:** Ionic liquids, Antimicrobial activity, Well-diffusion method, DFT, HOMO, LUMO and ADMET

## Abstract

The Brønsted acid-base neutralization was executed for synthesis of the anilinium carboxylate ionic liquids (ACILs), and obtained highly viscous liquids with yields about (90–94)%. These ILs were purified by distillation process and used vacuum oven, as well as characterized by FT-IR, UV spectroscopy and 1H-NMR. To evaluate the antimicrobial activity, the well diffusion method was used against eight human pathogenic bacteria, showing inhibition of zone at 13 mm–27 mm, and three fungi with result about 60%. Plus, the DFT functional from material studio 8.0 was used for evaluation of computational screening for estimating the chemical reactivity, HOMO, LUMO and HOMO-LUMO gap, recorded from -7.252 to -8.20 kcal/mol. The IL05 has showed about -6.5 kcal/mol docking score as standard inhibitor, as and higher than starting. Form AMDET properties, it has revealed that they have low toxicity, higher absorption through the biological system and non-carcinogenic. Finally, the electronegative groups, such as F, Cl and Br atoms in anion can show the higher antimicrobial activity and molecular docking score among all others while F atom containing IL05 shows the highest docking score and antimicrobial activity. However, it is concluded that rather than long large alkyl chain of anion, F atom (the highest electronegative atom) containing anion is better for biologically significance ILs.

## 1 Introduction

Ionic Liquids (ILs) have defined as the molted salts which consist of distinct anions and cations. The unique behaviors of ILs, for instance low melting point, large range of thermal stability, high volatility, negligible vapor pressure and tune-able physicochemical properties, makes them as ideal green solvent for the uses in chemical industries, pharmaceutical industries and research laboratories [1]. After commencement the area of ILs by Paul Walden in 1914, it had no sufficient development and research before 1980 [2]. After 1980, some prominent scientists, did thousands of highly significance research articles on ILs, and it had recognized as versatile materials for chemical industries, pharmaceutical industries and material engineering, as well as biological science for its tunable physical and chemical properties [3, 4, 5, 6]. Now a days, ILs has known as the engineering solvents for 21st century in consequence of designer properties and environmental sustainability [7]. The most common ILs is protic ILs which shows fascinating demands in numerous fields for its convenience appliances, such as activated carbon (AC) electrodes [8], magnifying ionic conductor [9], membrane in fuel cell [10], surfactant [11], biodiesel [12], fractionation of lignocelluloses biomass [13] and solvent for azeotropic mixtures [14]. Besides its gigantic applications, some of these are low toxic, obtainable and environmental benign materials with low cost [15]. Additionally, the synthesis of protic ILs has entirely followed the principle of green chemistry without losing solvents and high atom economic yields [16]. In general, their customized physiochemical properties lead them as the ideal aspirants to use in chemical industries, biological science, polymer chemistry, biochemistry and bio-process, chemical engineering, textile industries and catalytic chemistry [5,17,18]. ILs have also been starting as the alternative green solvents of traditional volatile organic solvents [19,20]. Withal, the protic ILs was being also used in the solvents for nucleophilic substitution reactions, polymerization processes [21], organic synthesis and biochemical process [22, 23, 24].

One of the most appealing features of protic ILs on pharmaceutical applications is quoted that they are highly customizable materials as bioactive molecule [25]. Last two decades, ILs have also attracted to the scientific community due to their pharmaceutical applications, such as antimicrobial, antiseptic or antifouling actions, delivery of anti-inflammatory drugs, anticancer activities and protein formulations [25, 26, 27]. Some of ammonium carboxylate ILs had been shown as bioactive molecule [28, 29, 30, 31] which was firstly reported by Whitney, L. *et al.* in 2009 [32]. Conversely, phosphonium and cholinium ILs are the most demanded chemicals for the uses of pharmaceutical purposes, particularly to design new drug. In case of toxicological study, it was found that the ammonium and phosphonium ILs are less toxic than all other classes of ILs. Regarding their low toxicity, the ammonium and phosphonium ILs had reported as potential anticancer molecules [33], microbial agents [34], antifungal agents [35], antibacterial agents [28,29], anti-biofilm molecules [36] and bio oil, lubricants or lubricant additives [37,38]. Due to those facts, this study has designed on ammonium cation ILs, particularly aromatic ring containing cation, has selected for the monitoring of their biological significance against human pathogenic micro pathogens. Plus, the effects of alkyl chain and electro-negative atoms in anion have been observed as a crucial aim through ILs molecules through the experimental study and with their mechanistic investigation using computational tools. However, the microbial activity of designed ammonium based ILs has not main attention but also elucidation of the alkyl chain and electronegative atom effect are going to the major concerns, which leads the obtained new desirable small molecules for pharmaceutical applications [25,39].

In addition, the experimental study of biological significance has comprised to the computational data obtained through the density functional theory (DFT) and molecular docking study which was placed the most accurate calculation for experimental and computational investigation [40,41]. The DFT method was executed to calculate the HOMO, LUMO, chemical descriptors and HOMO-LUMO gap, which have been considered as the chemical reactivity indicators of tested molecules [42, 43, 44, 45, 46]. To give more information about biological application, molecular docking study has done against eight ILs which provides information about their binding affinity to protein of pathogen, as well as a comparative docking score to show their binding ability with protein [47,48]. Finally, to evaluate the toxicity prediction about these ILs, the Absorption, Distribution, Metabolism, Excretion and Toxicity (ADMET) have accounted for their save uses as drugs.

## 2 Materials and methodologies

### 2.1 Materials and reagents

All used chemicals were research grade, and used without further purification, while solvents were also obtained by distillation before use.

The research grade chemicals were pursued for uses, and the solvents were distilled for obtaining more purification before use. The bacterial and fungal stains were collected from the department of Pharmacy in University of Dhaka and State University of Bangladesh, Bangladesh. The FT-IR spectrophotometer, (SHIMADZU, Japan, range 600–4500 cm-1) was used in the using of KBr disc technique. The 1H NMR spectra was recorded in Jahangirnagar University, Savar, Bangladesh. The synthesis, purification and analysis were completed at the department of chemistry in Bangladesh University of Engineering and Technology (BUET), Dhaka-1000, Bangladesh. The antibacterial and antifungal tests were performed at the department of Pharmacy in University of Dhaka, Bangladesh.

### 2.2 Synthesis and purification of ionic liquids

**Figure 1:**
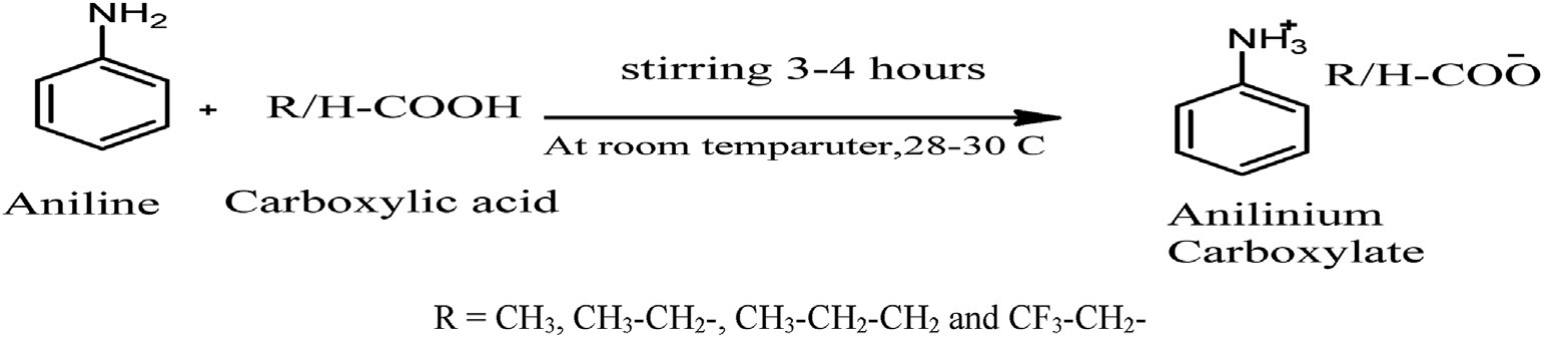
Synthesis reaction scheme.

The synthesis of anilinium carboxylate ILs was synthesized by an acid-base neutralization reaction [49]. The equimolar carboxylic acid (0.1 mol equivalent) was added into the round bottle containing aniline (0.1 mol equivalent) by dropwise during the time period of 20–25 min. The temperature of the reaction pot was maintained using ice-bath. Afterward, the mixture was stirred for (3–4) hours at room temperature until to obtain a viscous clear liquid. Initially, the reaction progress was monitored by thin layer chromatography (TLC). The main product of reaction was a salt of anilinium carboxylate which was viscous liquid or melted salt [49, 50, 51]. After reaction, the purification process was performed with a strong agitation and slight heating at 100 °C under pressure for removing impurities by vaporization. The pure ILs was obtained after this purification process, and the obtained liquids were looked a transparent and viscous façade state. The anilinium salt formation was characterized by analytical data obtained from FT-IR, 1H NMR and UV spectroscopy. The five ILs (Compound No. IL01, IL02, IL03, IL04 and IL05) were synthesized using the following reaction scheme shown in Figure 1.

### 2.3 Antimicrobial activity

#### 2.3.1 Preparation of IL solutions

The required amount of the ILs sample was measured for preparation of mili-Molar (mM) solution with high level of accurately so that no impurities were obtained. Moreover, the five various solutions, such as 1000 mM, 750 mM, 500 mM, 250 mM, and 125 mM, were prepared for determination of minimum inhibitor concentration (MIC).

#### 2.3.2 Antibacterial assay test

For a primary screening of antibacterial activity of pure ILs, the eight bacterial pathogens, such as *Bacillus cereus*, *Staphylococcus aureus*, *Sarcinalutea*, *Bacillus subtilis, and four are gram negative, such as Escherichia coli*, *Salmonella typhi*, *Pseudomonas aeroginosa* and *Shigella dysenteriae* were taken. The well diffusion method was carried on taking 20 μL of 1000 mM ILs solution in each well [52,53]. After completing the working progress for well diffusion method, the petri disc was kept in incubator at 36–37 °C for overnight for growth of bacteria strain. Then, the zone of inhibition (including the well diameter 8.0 mm) was measured in mm scale with thoughtfulness ±1.0 mm errors. This working progress was done in triple times, and the average magnitudes were listed in Table 1. It is noted that water was used as solvent to prepare ILs solution. As a result, water was considered as control.

**Table 1:** Zone of inhibition against bacteria in mm scale without 8 mm hole of well for 1000 mM.

	IL01	IL02	IL03	IL04	IL05	Control	Starting
*Bacillus cereus(+)*	18	20	21	18	24	0	0
*Staphylococcus aureus(+)*	16	17	19	14	25	0	0
*Bacillus subtilis(+)*	16	19	21	20	23	0	0
*Sarcina lutea(+)*	17	14	20	20	24	0	0
*Escherichia coli(-)*	16	14	21	18	19	0	0
*Salmonella typhi(-)*	14	16	15	27	27	0	0
*Pseudomona aeroginosa(-)*	13	14	17	13	19	0	0
*Shigella dysenteriae(-)*	16	14	17	17	20	0	0

#### 2.3.3 Antifungal assay test

The antifungal activity against three phytopathogenic fungi, such as *Aspergillus niger, Saccharomyces cerevisiae and Candida albicans* was as well performed by the well diffusion method. At first, 100 μL of ILs solution, 1000 mM, was added into petri plate, and then the agar media was added with well shaking so that the sample of ILs was mixed homogeneously with the media in petri plate. After solidification, the fungal strain was added in well and kept in incubator for 3 days maintaining temperature 36–37 °C. Then, the result was recorded in mm scale. The work was done triplet and taken the mean value listed in Table 2.

**Table 2:** Zone of inhibition of antifungal activity.

Chemicals tested	Zone of growth (in mm)			%, growth percentage		
	*Aspergillus niger*	*Saccharomyces cerevisiae*	*Candida albicans*	*Aspergillus niger*	*Saccharomyces cerevisiae*	*Candida albicans*
Control	28 mm	41mm	38 mm	100 %	100%	100 %
Staring material	28 mm	41mm	38 mm	100 %	100 %	100 %
IL01	15	19	17	53.00 %	46.34%	44.70%
IL02	14	17	16	50.00 %	41.46%	42.10%
IL03	13	17	15	46.43 %	41.46%	36.58%
IL04	11	16	13	39.28%	39.02%	34.21%
IL05	12	15	13	42.85%	36.58%	34.21%

#### 2.3.4 Test for minimum inhibition concentration (MIC)

The MIC test was evaluated by well diffusion method for five concentration (1000 mM, 750 mM, 500 mM, 250 mM and 125 mM) against *Bacillus cereus(+)* and *Escherichia coli*(+) using similar the assay of anti bacterial test and recorded the data and analysis these data listed in Table 4.

### 2.4 Computational details for molecular optimization

The material studio 8.0 was used for molecular modeling and theoretical investigation of ILs [54]. The optimization of molecules was usually done using the DFT functional with basis set of B3LYP from the DMol3 code of material studio setting the criteria of ground state, unrestricted spin, minimum basis set, 3.5 basis files and fine cut off [40]. After optimization, the value of HOMO, LUMO and molecular orbital of HOMO, LUMO were calculated from the optimized structure.

It is crucial note that IL06, IL07 and IL08 were designed by molecular modelling and computational approaches for calculating their biological studies via computational tools. Besides, it has illustrated that this studies does not complete without these compounds so that it has designed for evaluating the biological approaches on the anion chain and effect of electronegative atoms. The molecules, IL06, IL07 and IL08, imply to named aniliniumpentanoate, aniliniumtricloroaccetate and aniliniumtribromoaccetate, respectively.

### 2.5 Molecular docking

For molecular docking, the ILs as ligands were optimized using DMol3 code (DFT, B3LYP) and save as protein data bank (pdb) file. One fungi, *Aspergillus niger* (PDB id: 1kum), and two bacteria, *Bacillus cereus(+),(PDB ID: 5v8d)* and *Escherichia coli(-) (PDB ID: 3ch3),* crystal structure of proteins were taken for performing molecular docking from RSCB Protein Data Bank [55]. The three crystal of proteins were optimized and checked by PyMol (version 1.1) software package, and all the hetero atoms, water molecules and contaminated inhibitor were removed from raw protein for obtaining fresh protein [56]. The Autodock Vina was used for molecular docking [57]. Eventually, the Discovery Studio 4.1 Client was used for the visualization of binding modes of the receptor ligands interaction [58].

### 2.6 Evaluation of ADMET and Lipinski's rule

To calculate approximately the drug related indicators (absorption, distribution, metabolism, excretion and toxicity) for ILs were calculated employing admetSAR (http://lmmd.ecust.edu.cn/admetsar2/) online database [59]. On the other hand, Lipinski's rule of five, which is the one of formula to become drugs, was calculated the other online portal named SwissADME (http://www.swissadme.ch/^)^ [60].

## 3 Results and discussions

### 3.1 Characterization

The chemical shift from 1H NMR spectrum for Aniliniumtrifluroaccetate (IL05) was obtained at 7.981 (s, 3H, PhNH_3_), 7.21 (t, 2H, Ph), 6.71 (d, 3H, Ph). There was not found any chemical shift at 3.55 ppm which was characteristic peak for –NH_2_ group, and disappeared the peak at 11.42 ppm which was obtained for –COOH group. However, the absence of –COOH and –NH_2_ group indicated the conversion of amine and carboxylate groups in ammonium carboxylate ILs. In case of FTIR, the strong peaks at about 3429 cm^−1^ (N–H) asymmetry and 3004 cm^−1^ (N–H) symmetry provide the presence of ammonium ion [61], as well as the another two peaks at 1780 cm^−1^ (C–O) asymmetry, 1675 cm^−1^ (-CO) symmetry confirm the existence of carboxylate ion [62]. However, the almost similar FTIR peaks were obtained for IL01, IL02, IL03 and IL04. Withal, the UV spectra give the similar absorption at about 240 nm wavelength which is almost same for all ILs.

**Anilinium methnoate (IL01),** [PhNH_3_] [C_1_OO], M.W.:138, Yield (%): 91.0%

FT-IR (KBr) in cm^−1^: 3277 (N–H) asymmetry, 3087 (C=C) in benzene ring, 2967 (N–H) symmetry, 2934 (C–H) asymmetry, 2854 (C–H) symmetry, broad peak in 2370 (PhNH_3_^+^), 1712 (C–O) asymmetry and 1682 (-CO) symmetry, cm^−1^.

**Anilinium ethanoate (IL02),** [PhNH_3_] [C_2_OOH_3_], M.W.:153, Yield (%): 93.0%

FT-IR (KBr) in cm^−1^: 3358 (N–H) asymmetry, 3217 (C=C) in benzene ring, 3037 (N–H) symmetry, 2908 (C–H) asymmetry, 2897 (C–H) symmetry, broad peak in 2640 (PhNH_3_^+^), 1681 (C–O) asymmetry, 1621-1602 (-CO) symmetry, cm^−1^.

**Anilinium propanoate (IL03),** [PhNH_3_] [C_3_OOH_5_], M.W.: 167.0, Yield (%): 91%.FT-IR (KBr) in cm^−1^: 3334 (N–H) asymmetry, 3087 (C=C) in benzene ring, 3199 (N–H) symmetry, 2977 (C–H) asymmetry, 2910 (C–H) symmetry, 2697 (PhNH_3_^+^), 1667 (C–O) asymmetry, 1603 (-CO) symmetry, cm^−1^.

**Anilinium butanoate (IL04),** [PhNH_3_] [C_4_OOH_7_], M.W.: 187.0, Yield (%): 91%. FT-IR (KBr) in cm^−1^: 3287 (N–H) asymmetry, 3000 to 3050 (C=C) in benzene ring, 3197 (N–H) symmetry, 2874 (C–H) asymmetry, 2803 (C–H) symmetry, 2646 (PhNH_3_^+^), 2352 (C–O) asymmetry, 1660 (-CO) symmetry, cm^−1^.

**Aniliniumtrifluroaccetate (IL05),** [PhNH_3_] [C_2_F_3_OOH_3_], M.W.: 207.0, Yield (%): 98%. FT-IR (KBr) in cm^−1^: 3429 (N–H) asymmetry, 3004 (N–H) symmetry, 2800 (C–H) symmetry, 2596 (PhNH_3_^+^), 1780 (C–O) asymmetry, 1675 (-CO) symmetry, cm^−1^.

^1^H-NMR chemical shifts (400 MHz, D_2_O): 7.981 (s, 3H, PhNH_3_), 7.212 (t, 3H, Ph) and 6.718 (d, 3H, Ph).

### 3.2 Biological studies

#### 3.2.1 Antibacterial studies and effect of anion

The Table 1 represents the zone of inhibition against bacteria which was measured by well diffusion method, and the result was taken by average from triplet test. The control was used as solvent, water, in which the ILs was dissolved for making solution, and it shows the zero activity in same concentration. On the other hand, aniline was starting material which had no result on antibacterial test.

**Figure 2:**
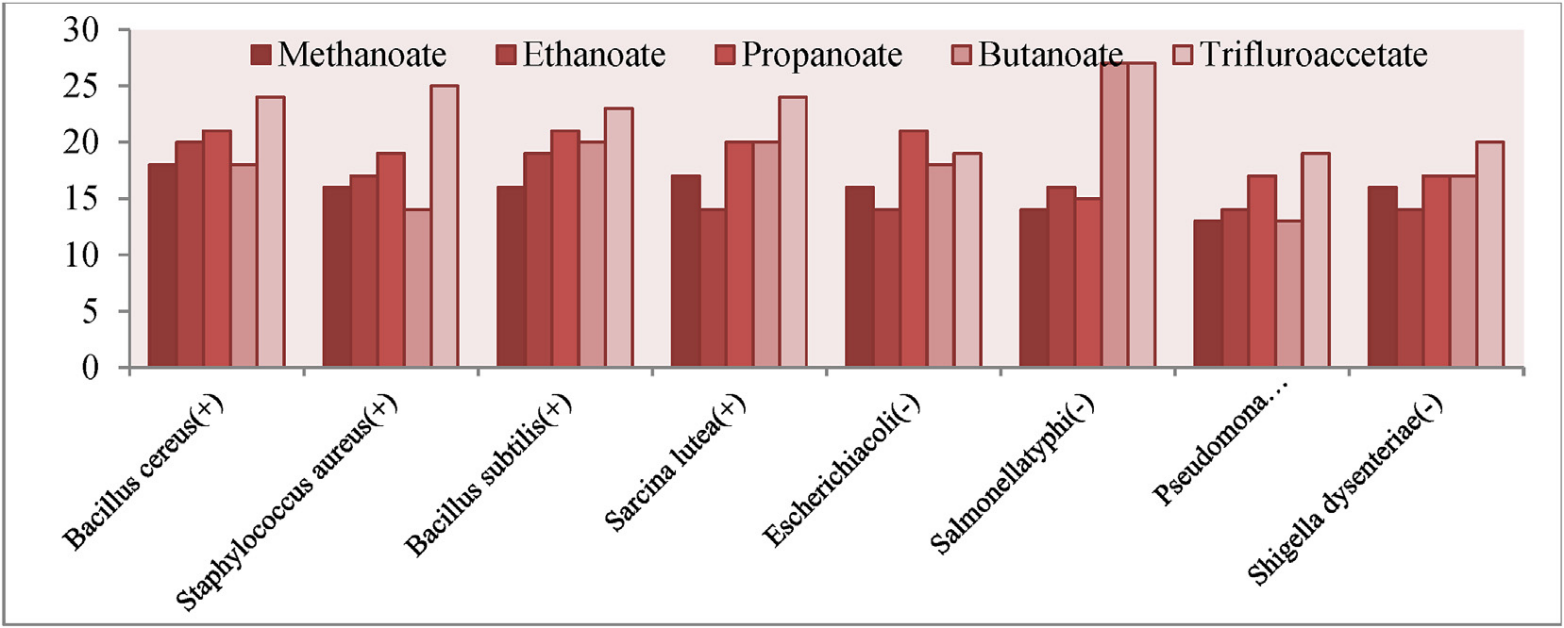
The relative effect of anion on antibacterial activity.

In case of antibacterial activity, the effect of anion was estimated using the methanoate, ethanoate, propanoate, butanoate and trifluroethanoate anion. For the each bacteria pathogen, the anion has to be shown an effect on antibacterial activity. From the Figure 2, the trifluroethanoate anion can show the highest activity among methanoate, acetate, propanoate and butanoate against both gram positive and gram negative bacteria whereas the gram positive bacteria shows the slightly more response than gram negative bacteria. Moreover, it can be revealed that with increasing the alkyl chain, the zone of inhibition is slowly increased although it has found the highest activity due to fluorine atom attaching in anion.

#### 3.2.2 The antifungal activity result

The antifungal test was completed through well diffusion method in which the growth of inhibition for both control and synthesized ILs. The growth percentage of ILs was calculated compared with the growth percentage of control where the growth of control is 100% percent. The growth percentage is deduced as the following equation:

$$\text{\%}\;\text{Growth}\;\text{percentage}=\frac{\text{Growth of fungi with ILs solution}}{\text{Growth}\;\text{of}\;\text{fungi}\;\text{without}\;\text{ILs}\;\text{solution}\;\text{as}\;\text{control}}\times 100$$

#### 3.2.3 Calculation of percentage of inhibition and effect of anion on antifungal activity

The percentage of inhibition is calculated by following equation

Percentageofinhibition=( Growth percentageofcontrol)−(Growth percentage of ILs sample)

In view of antifungal activity, the effect of anions (methanoate, acetate, propanoate, butanoate and trifluroacetate) was carried on through the percentage of inhibition which indicates the ability to kill the fungal by drugs. Withal, the large value of percentage of inhibition illustrates the higher active molecule against fungal pathogens. The three phytopathogenic fungal were conducted to the evaluation on antifungal activity, as well as effect of anions shown in Table 3. The butanoate anion can show the higher activity among methanoate, acetate, trifluroacetate and propanoate anion of ILs for *Aspergillus niger*, but the trifluroacetate anion (IL05) can also show the highest antifungal activity among others against *Saccharomyces cerevisiae* and *Candida albicans*. The IL05, containing Fluorine atom, can show the highest antifungal activity although IL04 is close to its magnitude.

**Table 3:** Data for percentage of inhibition.

Chemicals tested	Percentage of Inhibition		
	*Aspergillus niger*	*Saccharomyces cerevisiae*	*Candida albicans*
Control	0%	0%	0 %
Staring material	0%	0%	0%
IL01	47.00%	53.66%	55.30%
IL02	50.00%	58.54%	63.43%
IL03	53.58%	58.54%	55.30%
IL04	60.72%	60.98%	65.79%
IL05	57.15%	63.42 %	65.79%

#### 3.2.4 Calculation of MIC

The MIC for the tested ILs were calculated from the inhibitions showed in the different concentrations via serial dilution, such as 1000 mM, 750 mM, 500 mM, 250 mM, and 125mM using the well diffusion technique. For MIC studies, two bacterial pathogens, for instance *Bacillus cereus (+) and Escherichia coli (-)* were chosen, because they are common human pathogenic microorganism and obtained the highest antibacterial activity for primary screening. From the Table 4, it was found that the MIC for each ILs against all bacteria from 250 mM to 125 mM. For both *Bacillus cereus (+) and Escherichia coli (-),* the MIC was recorded minimum for IL05.

**Table 4:** MIC.

*Bacillus cereus*(+)						
	1000 mM	750 mM	500 mM	250 mM	125 mM	MIC
IL01	18	11	6	0	0	250
IL02	20	16	9	0	0	250
IL03	21	14	7	0	0	250
IL04	18	10	6	0	0	250
IL05	24	16	11	7	0	125
*Escherichia coli(-)*						
IL01	16	10	5	0	0	250
IL02	14	7	0	0	0	500
IL03	21	19	9	0	0	250
IL04	18	11	6	0	0	250
IL05	19	13	9	6	0	125

### 3.3 Computational studies

#### 3.3.1 HOMO and LUMO

**Figure 3:**
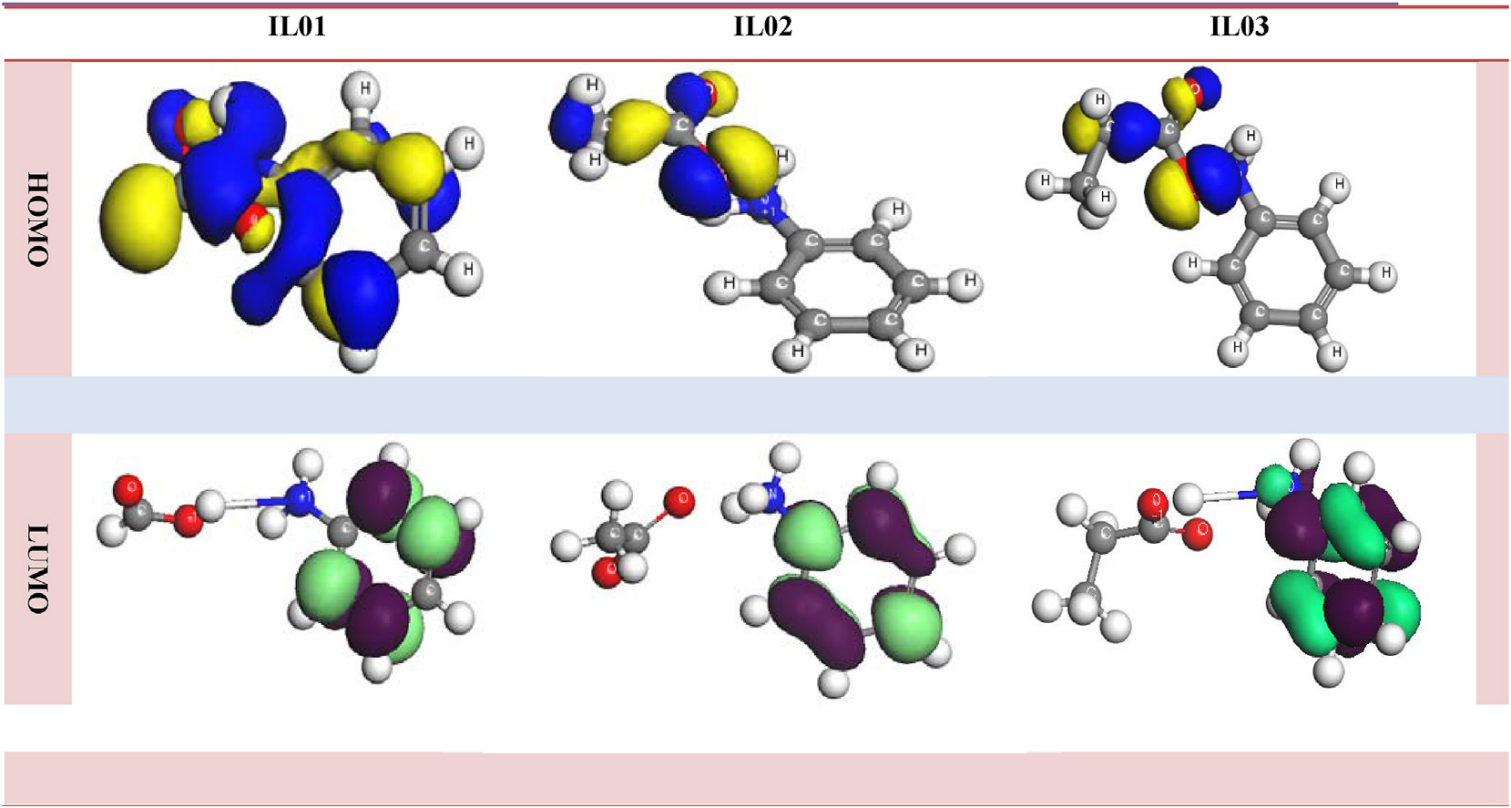
Frontier orbital diagram of HOMO LUMO.

**Figure 4:**
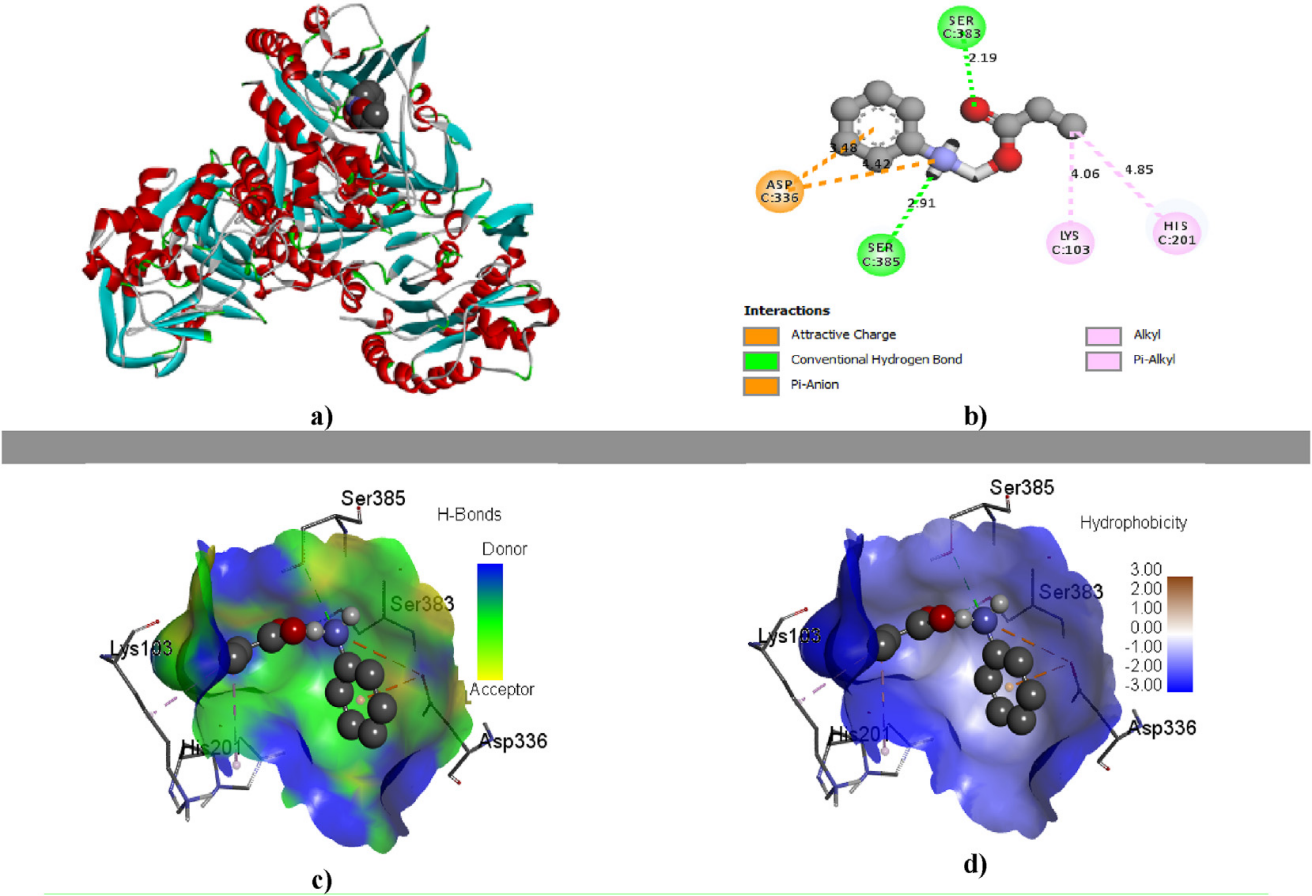
a) Ligand in protein pocket b) 2D picture of ligand protein interaction c) Hydrogen bonding pose of ligand protein d) Hydrophobicity; of IL03 against *Bacillus cereus* (+).

The term, HOMO, implies to highest occupied molecular orbital, and LUMO belongs to lowest unoccupied molecular orbitals which are also well thought-out substantial orbitals of frontier molecular orbitals (FMOs). In term of quantum mechanism, HOMO demonstrates the valance band electrons which contain highly denser electrons where the electrophilic groups can be attracted for adding by forming weak chemical bonding. On contract, the LUMO is equal to conduction band, and illustrates the attraction of nucleophilic groups. Overall, the HOMO-LUMO gap of molecules indicates the chemical stability and chemical reactivity, as well as biological activity as drugs has to find. From the Figures 3 and 4(S), it can be illustrated that how a ligand or drug is to be bonded with protein of pathogens through various orientations in terms of HOMO and LUMO sites of ILs.

• Blue color is the positive node, and yellow is the negative node for HOMO.
• Parrot color is positive, and violet color is the negative node for LUMO.
• The most notation finding is noted that the LUMO for all molecules put down in aromatic ring of cation.
• The HOMO is located through the anion, and most of parts HOMO are in carboxylate ions where the oxygen atoms have attached.

Moreover, the energies of FMOs are the most useful tools to calculate the chemical reactivity descriptors, for instance global softness (S), ionization potential (I), electron affinity (A), electronegativity (χ), hardness (η), electrophilicity index (ω) and chemical potential (μ) utilizing given equations [63]. The Koopmans's theorem was used to calculate the chemical potential, electronegativity, and hardness, besides the softness and electrophilicity index were evaluated by Parr *et al.* [63] using equation no (04–08). According to Koopmans' theorem, the LUMO-HOMO gap (Egap) commonly known as the energy gap, defined as the difference the energy from LUMO to HOMO [64] from equation no 01, and ionization potential and electron affinity were calculated by Eqs. (02) and (03), respectively.

(1) $${\text{E}}_{\text{gap}}=\left({\text{ E}}_{\text{LUMO}}-{\text{E}}_{\text{HOMO}}\right)$$

The ionization potential (I) and electron affinity (A) can be estimated from the HOMO and LUMO energy values as following

(2) $$I=-E_{HOMO}$$

(3) $$A=-E_{LUMO}$$

Some chemical descriptors, such as electrophilicity, chemical potential, electronegativity, hardness and softness are calculated as following equations and listed in Table 5:

(4) $$\left(\mu \right)=-\frac{I+A}{2}$$

(5) $$\left(\eta \right)=\frac{I-A}{2}$$

(6) $$\left(\text{σ}\right)=\frac{1}{\eta }$$

(7) $$\left(\chi \right)=\frac{I+A}{2}$$

(8) $$\left(\omega \right)=\frac{\mu^{2}}{2\eta }$$

➢ The energy gap or LUMO-HOMO gap is about below 8.0 for all except IL05 and IL07.
➢ The LUMO-HOMO gap for IL05 and IL07 are 8.303 and 8.200 eV while IL05 shows the highest gap.
➢ IL05 has the highest hardness value (4.152 eV).
➢ As IL05 shows the lowest softness among all ILs, it can be said that it is highly biological active, and IL05 dissociates more easily among others.
➢ Finally, fluorine atoms on anion (IL05) as electronegative group are highly impressed for chemical reactivity as well as biological dissociation study through the term of energy gap.

**Table 5:** HOMO, LUMO, HOMO–LUMO gap, Ionization potential (I) and Electron affinity.

	c	IL02	IL03	IL04	IL05	IL06	IL07	IL08
HOMO, eV	-8.582	-7.332	-8.434	-8.443	-9.485	-8.453	-9.351	-7.450
LUMO, eV	-0.601	-1.594	-0.732	-0.736	-1.181	-0.910	-1.151	-1.947
LUMO-HOMO gap, eV	7.981	5.738	7.702	7.713	8.303	7.543	8.200	5.503
Ionization potential(I), eV	8.582	7.332	8.434	8.443	9.485	8.453	9.351	7.450
Electron affinity (A), eV	0.601	1.594	0.732	0.736	1.181	0.910	1.151	1.947
Chemical potential (μ), eV	4.591	4.463	4.583	4.589	5.333	4.681	5.251	4.698
Electrophilicity index(ω), eV	2.641	3.471	2.727	2.734	3.425	2.905	3.362	4.011
Electronegativity ), eV	-4.591	-4.463	-4.583	-4.589	-5.333	-4.681	-5.251	-4.698
Hardness (η), eV	3.990	2.869	3.851	3.853	4.152	3.771	4.100	2.751
Softness (σ), eV	0.250	0.348	0.259	0.259	0.240	0.265	0.244	0.364

#### 3.3.2 Molecular docking with docking score

The focal task of docking study has employed to investigate the possible interaction site evaluation between ligands (ILs) and macromolecules, such as *Bacillus cereus (+), Escherichia coli (-), and Aspergillus niger (fungi),* showing the all diagram against pathogens through the terms as ligands in protein pocket, hydrogen bonding, hydrophobicity and 2D diagram in Figure 4, as well as their binding affinity with these pathogens are given in Table 6. It already was established that the magnitude of binding affinity for standard drug is almost 6.0 kcal/mol or above [65,66]. From the Table 6, it is shown that the starting material named aniline can show very poor binding affinity which is about 3.0 kcal/mol. To make and prepare eight different anilinium carboxylate ILs, eight different carboxylic acids were used with aniline base where it was found from molecular docking score and H bonding affinity, showing in Figure 4 and Table 6, that the binding score has growth up to reaching the standard drugs score. From the Table 6, it is noted that IL05 illustrates the highest binding affinity, which is more than 6.0 kcal/mol against three pathogens and IL07 and IL08 are next molecules in term of molecular docking score by binding affinity. From the full analysis of Table 6, accounting the binding affinity, it can be summarized that alkyl chain of anion has a regular effect for binding with pathogens of protein while fluorine atoms on anion (IL05) can show the highest binding affinity forming by halogen bonds.

**Table 6:** Docking score by interaction between ligand and macromolecule.

	*Bacillus cereus*(+)			*Escherichia coli*(-)			*Aspergillus niger*(Fungi)		
	Binding affinity (kcal/mol)	No of H bond	Total bonds	Binding affinity (kcal/mol)	No of H bond	Total bonds	Binding affinity (kcal/mol)	No of H bond	Total bonds
IL01	-5.9	04	05	-5.8	03	04	-5.4	03	05
IL 02	-6.6	02	04	-6.1	02	07	-5.8	01	05
IL 03	-6.0	02	04	-5.8	02	07	-6.0	04	05
IL 04	-5.8	03	08	-5.5	02	06	-6.0	03	04
IL 05	-6.7	03	06	-6.0	03	08	-6.5	02	06
IL06	-5.5	00	03	-5.9	01	03	-6.2	02	04
IL 07	-6.2	04	05	-6.0	03	06	-6.3	03	04
IL08	-6.0	02	04	-5.8	00	04	-6.4	03	05
Starting	-2.8	00	03	-3.0	00	03	-2.8	00	04

Secondly, from the orbital picture of H bonding, hydrophobicity, 2D diagram and molecular interaction, it is clear that the hydrogen bonding and charging attraction with protein are occurred for binding as drug presented in Figure 4. In case of ILs, the charging attraction showing Figure 4, which is special case from other organic compounds and additional score, is illustrated with protein. Moreover, hydrogen-bonds and charging attraction execute a vital function in shaping the specificity of ligand binding with the receptor in process of drug design in chemical and biological system, molecular identification and biological activity. From the 2D diagram, it is crystal clear that the H-binding is formed by both cation and anion parts with protein that is not varied causes for molecular docking score. From Figure 3 in terms of the HOMO and LUMO picture, cation indicates the LUMO position which implies the both of H bonding and charging attraction. The nitrogen atom of ammonium ion, which is attached with benzene ring, and pi electron of benzene ring make to lead the charging attraction bond with protein. In general, the oxygen atom of anion can be able to make the conventional H bond with protein, and the alkyl chain of anion is to response forming the alkyl pi bond as H bond.

### 3.3.3 Physiochemical properties and Lipinski rule

In general, to evaluate the physiochemical properties and Lipinski rule, the online database, SwissADME, was used, over and above predicted drug-likeness and pharmacokinetics properties, which are decisive tools for drug design. It is generally estimated the common fact for drug-likeness which is derived by Lipinski's rule of five [67]. From the Table 7, all listed ILs are satisfied by Lipinski rule, besides they are recognized the high GI absorption and almost similar skin permeation.

**Table 7:** Docking score by interaction between ligand and macromolecule.

Compounds	*Bacillus cereus(+), (5v8d)*			*Escherichia coli(-), (3ch3)*			*Aspergillus niger(Fungi), (1ks5)*		
	Amino acid residue	Bond distance	Bond type	Amino acid residue	Bond distance	Bond type	Amino acid residue	Bond distance	Bond type
IL01	LYS = 110	4.15	Pi alkyl	SER-21	3.01	H bond	ASN-63	2.6	H bond
	SER-388	2.45	H bond	GLY-385	2.80	H bond	ASN-20	2.73	H bond
	LUE-387	2.35	H bond	GLU-382	2.43	H bond	GLN-200	2.10	H bond
	ASN-386	3.60	H bond	ALA-18	3.81	Pi sigma	ASP-99	5.48	Attractive charge
	ASN-386	5.15	C– H bond				TRP-22	6.82	Pi-pi stacked
IL 02	LEU-387	2.52	H bond	ARG-386	2.04	H bond	ASN-20	2.74	H bond
	SER-388	2.59	H bond	SER-14	3.21	H bond	ASN-63	2.56	H bond
	LYS-110	4.08	Pi alkyl	MET-30	5.42	Pi alkyl	GLN-200	2.13	H bond
	LEU-387	5019	Pi alkyl	VAL-15	5.12	Pi alkyl	GLN-200	2.18	H bond
				LEU-197	4.96	Pi alkyl	TRP-22	6.84	Pi alkyl
				GLU-483	2.48	Attractive charge			
				GLU-483	3.04	Salt bridge			
IL 03	SER-383	2.19	H bond	ARG-386	2.06	H bond	ASN-63	2.76	H bond
	SER-385	2.91	H bond	SER-14	3.10	H bond	GLN-300	2.56	H bond
	ASP-336	3.48	Pi anion	GLU-483	2.92	Attractive charge	ASN-20	2.11	H bond
	ASP-336	4.42	Attractive charge	GLU-483	2.44	Salt bridge	GLN-300	2.22	H bond
	LYS-103	4.06	Pi alkyl	VAL-15	5.12	Pi alkyl	REP-22	6.84	Pi alkyl
	HIS-201	4.85	Pi alkyl	LEU-197	5.00	Pi alkyl			
				MET-30	3.85	Pi alkyl			
IL 04	SER-385	2.81	H bond	SER-14	2.67	H bond	ASN-63	2.57	H bond
	SER-383	1.96	H bond	SER-14	2.43	H bond	GLN-200	2.11	H bond
	ASP-336	3.48	Pi anion	GLU-483	1.17	Salt Bridge	GLN-200	2.23	H bond
	ASP336	4.63	Attractive charge	MET-30	4.94	Pi alkyl	TRP-22	6.83	Pi-pi stacked
	LYS-103	5.02	Pi alkyl	MET-30	4.82	Pi alkyl			
	HIS-201	5.09	Pi alkyl	LEU-197	4.11	Pi alkyl			
	LYS-143	4.25	Pi alkyl						
	SER-142	3.16	C–H bond						
IL 05	PHE-135	2.08	H bond	SER-14	3.31	H bond	ASN-20	2.75	H bond
	PHE-135	2.22	H bond	ARG-386	2.06,2.86	H bond	ASN-20	2.18	H bond
	ASP-173	2.94	H bond	GLU-483	2.55,2.85	Salt Bridge	GLN-200	3.02	Halogen bond
	GLU-134	3.68	Halogen bond	LEU-197	4.97	Pi alkyl	SER-111	3.29	Halogen bond
	GLU-134	3.46	Halogen bond	MET-30	5.39	Pi alkyl	ASP-99	3.41	Halogen bond
	ASP-173	4.75	Attractive charge	VAL-15	5.23	Pi alkyl	ASP-99	2.97	Halogen bond
IL06	ASP-293	3.17	Salt Bridge	SER-14	2.59,2.35	H bond	GLN-200	2.23,2.14	H bond
	TYR-267	4.19	Pi alkyl	GLU-483	2.21,2.41	Salt Bridge	ASN-63	2.56	H bond
	LEU-292	5.41	Pi alkyl	MET-30	5.68	Pi anion	TRP-22	6.92	Pi-pi stacked
							TYR-07	4.78	Pi alkyl
IL 07	PHE-276	2.09	H bond	SER-14	2.64,2.58	H bond	ASN-20	2.89	H bond
	PHE-276	2.14	H bond	ARG-386	2.43	H bond	ASN-63	2.07	H bond
	ASP-372	2.70	H bond	GLU-483	4.49	Attractive charge	GLN-200	2.17	H bond
	ASP-372	2.62	H bond	VAL-15	5.13	Pi alkyl	TRP-22	6.51	Pi-pi stacked
	ASN-275	5.27	Pi-pi stacked	LEU-197	4.60	Pi alkyl			
IL08	GLN-348	2.11	H bond	GLU-483	2.40	Salt Bridge	GLN-128	2.30,2.09	H bond
	GLN-348	2.37	H bond	ARG-386	3.51	Salt Bridge	GLY-56	3.68	C–H bond
	ALA-325	5.22	C–H bond	MET-30	5.70	Pi anion	PHE-206	4.91	Pi-pi stacked
	GLN-326	4.89	Pi alkyl	ALA-18	4.62	Pi alkyl	ILE-130	5.37	Pi alkyl

The lipophilicity, which is determined by partition-coefficient of a drug in octanol and water medium, and it is denoted by Log Po/w, is an important parameters for a drug-like molecule. Overall, the most standard value of lipophilicity is -0.7 and +5.0. From the Table 8, it is visibly listed that all ILs maintains the standards value which is from +0.06 to +1.21. Among of them, IL06 and IL08 have higher magnitude than 1.0 while others stay below than 1.0. In term of TPSA, all values are near to 40.0 without IL01 and IL05 which value is about 64.0. In sum up, it can be written up that on physiochemical properties, alkyl chain has an effect though flurine atoms attaching in anion show the highest outcome on biological activity.

**Table 8:** Data of Physiochemical properties and Lipinski rule.

	MW	NRB	HBA	HBD	TPSA	Lipophilicity Log Po/w	Log Kp (skin, permeation) cm/s	Lipinski rule		GI absorption
								Score	violation	
IL01	140.16	0	3	2	64.94	0.08	-6.66	Yes	0	High
IL 02	154.19	0	3	2	40.54	0.06	-6.75	Yes	0	High
IL 03	168.21	2	3	2	40.54	0.40	-6.50	Yes	0	High
IL 04	182.24	2	3	2	40.54	0.74	-6.33	Yes	0	High
IL 05	208.16	1	6	2	64.94	0.88	-6.28	Yes	0	High
IL06	196.27	3	3	2	40.54	1.10	-5.63	Yes	0	High
IL 07	257.52	1	3	2	40.54	0.91	-6.07	Yes	0	High
IL08	390.87	1	3	2	40.54	1.21	-6.52	Yes	0	High

Note: MW: molecular weight, HBD: No. of H-bond donors, HBA: No. of H-bond acceptor, NRB; No. of rotatable bonds, TPSA: topological polar surface area.

#### 3.3.4 Pharmacokinetics and ADMET studies

Being ionic and charge compounds used in this study, it is open a door of the toxicity for both of aquatic and non aquatic ecology. Regarding this prospective, there is a tremendous risk for human health, as well as plant and aquatic living organisms when it will be mixed into environment after use. Keeping these scenes, there has developed a theoretical investigation for used molecules through the ADMET studies which was performed via the online portal named as AdmetSAR, and it implies to the line http://lmmd.ecust.edu.cn/admetsar2/.

The Table 9 represents the data of ADMET properties including human intestinal absorption, human oral bio-availability, blood brain barrier, P-glycoprotein substrate/inhibitor, sub-cellular localization, CYP substrate and inhibitor, carcinogenicity and aquatic and non aquatic toxicity. All ILs are positive to human intestinal absorption and blood brain barrier while negative responses are noted form Table 9 for the terms of human intestinal absorption, human oral bio-availability, blood brain barrier, p-glycoprotein substrate/inhibitor, sub-cellular localization, CYP substrate and inhibitor. Although, they have negative result on carcinogenicity which makes them as the most useful materials, and there are found positive result on both of aquatic and non aquatic living organisms. In case of fish toxicity, IL04 gives the highest score than others, as well as it is changed with increasing alkyl chain though the longest alkyl chain attached in IL06. On the other hands, it has changed on basis of alkyl chain in term of solubility whereas the highest solubility is found in IL06 although it has as well influenced by electronegative atoms. Regarding this fact, the highest solubility among all used molecules is for box of IL08 which contains the value 2.90. The main reason is noticed for its attaching halogen atoms where the large size bromine atoms are presented. It is concluded that the toxicity of used ILs is changed due to both of alkyl chain and electronegative atoms which halogen atoms are highly impressed the toxicity than alkyl chain on anion.

**Table 9:** Pharmacokinetic parameters of photochemicals.

Drug Candidate	Human Intestinal Absorption (+ve/-ve)	Human oral bioavailability (+ve/-ve)	Blood Brain Barrier (+ve/-ve)	P-glycoprotein substrate/inhibitor	Subcellular localization	CYP substrate	CYP Inhibitor	Carcinogenicity (binary) (+ve/-ve)	Fish aquatic toxicity (+ve/-ve)	Water solubility Logs	Acute Oral Toxicity (kg/mol)	Tetrahymena pyriformispIGC50 (ug/L)
IL01	+	-	+	−/−	Mitochondria	-	-	-	0.746	-1.138	2.657	0.284
IL 02	+	-	+	−/−	Mitochondria	-	-	-	2.782	-1.854	1.842	-0.266
IL 03	+	-	+	−/−	Mitochondria	-	-	-	2.510	-1.847	1.972	0.171
IL 04	+	-	+	−/−	Mitochondria	-	-	-	2.121	-2.038	2.217	0.273
IL 05	+	-	+	−/−	Mitochondria	-	-	-	1.632	-2.200	2.545	-0.071
IL06	+	-	+	−/−	Mitochondria	-	-	-	1.894	-2.490	2.044	0.315
IL 07	+	-	+	−/−	Mitochondria	-	-	-	1.473	-2.882	2.215	0.192
IL08	+	-	+	−/−	Mitochondria	-	-	-	1.681	-2.928	2.224	0.436

#### 3.3.5 Interaction of amino acid residue with ILs

The Table 7 represents about the data which is derived from the 2D diagram of various bond interactions between amino acid residues of protein and ILs as ligands as well types of bonding. Moreover, it illustrates the bond distance between amino acid residues and ILs which also indicates about the stability of bonding and how energy is required to break down the bond. For example, it might be thought that smaller bond distance indicates the stronger attraction which is upshot the molecular docking score.

In this study, the most noteworthy factor is noted for ILs on biological interaction or biological affection by molecular docking study which provides information about biologically significance, and how they shows more affection to bind with protein as antimicrobial agent or antibiotic regarding as the mechanistic study. From the Table 7 and Figure 4, it is found that some new bonds or interaction, such as attractive charge, salt bridge and pi anion bond have obtained which are not formed for normal organic molecules. That is why the ammonium ion or such type of ions in ILs can contribute to form these bonds with protein which leads to enhance the molecular docking score. The one of the main novelty of the computational part of this study is that it has picked up and introduced how cation part of ILs gives the guide about charging attraction with protein, as well as the negative part of anion illustrates the hydrogen bond which is the strongest bond, and distance is about 2.00 A° in maximum cases among all other bonds. In case of IL05, it introduces the extra halogen bond with minimum bond distance which gives it highest docking score although IL07 and IL08 have not produced the halogen bond having chlorine and bromine atoms. Because chlorine and bromine are not much electronegative to produce the halogen bond with protein compared to fluorine which is attached in IL05. Therefore, it is transparently clear that the fluorine atom can form the halogen bond with protein not other halogen atoms, because it has high electronegativity like oxygen atom which leads to show the high biological activity.

## 4 Conclusion

The synthesis of anilinium carboxylic ILs was carried on without any solvent media that indicates the way of green chemistry criteria. Just about 3–4 h stirring of the required acid base mixture, ILs was obtained (90–94) % yield and it was comparatively simple technique for the purification by rotary distillation. The analytical data of IR, NMR and UV give the superior supports for confirmation of reaction and structure of molecules. To estimate the bioactivity of ILs, although the antibacterial activity of starting material showed as poor inhibitor against all taken pathogens, the synthesized ILs was showed the 13–27 mm zone of inhibition against bacteria. Secondly, the antifungal activity was estimated more than 50% as zone of inhibition where IL05 can show more than 60% of percentage of inhibition. More specific data for bacterial pathogens, the MIC was performed against two pathogens, such as *Bacillus cereus (+) and Escherichia coli (-)* from where it is noted that the MIC value remains between 250 to 125 mM/L. In addition, for evaluation of computational investigation using computational data, the chemical reactivity in views of HOMO- LUMO gaps are varied on based of both alkyl chain and electronegative groups. The energy gaps for IL01 to IL08 are 5.50–8.30 kcal/mol. Among of them, IL05 shows the highest energy gap and hardness while IL06 shows it opposite trends. Moreover, IL05 illustrates the lowest softness which indicates the most affection to biological activity. The similar tendency has found for molecular docking score, ADMET and Lipinski rule for IL05. The IL05 illustrates the highest molecular docking score which is explained one specific reason that it has highly electronegative atom (Fluorine atom in anion) which can form the extra halogen bonds and charging attractive bonds with macromolecules. From this study in view of antimicrobial and computational approaches, it can be noted that both of alkyl chain and electronegative atoms on anion have a variable effect on biological activity, but the effect of electronegative atoms on anion is highly preferred as drugs than alkyl chain.

## Declarations

### Author contribution statement

Ajoy Kumer: Conceived and designed the experiments; Performed the experiments; Analyzed and interpreted the data; Contributed reagents, materials, analysis tools or data; Wrote the paper.

Md. Wahab Khan: Conceived and designed the experiments; Contributed reagents, materials, analysis tools or data.

### Funding statement

This work was supported by Bangladesh University of Engineering and Technology (10.13039/501100009500BUET), Dhaka-1000, Bangladesh.

### Data availability statement

No data was used for the research described in the article.

### Declaration of interests statement

The authors declare no conflict of interest.

### Additional information

No additional information is available for this paper.
